# Detecting Heart Failure Decompensation by Measuring Transthoracic Bioimpedance in the Outpatient Setting: Rationale and Design of the SENTINEL-HF Study

**DOI:** 10.2196/resprot.4899

**Published:** 2015-10-09

**Authors:** Silviu Dovancescu, Jane S Saczynski, Chad E Darling, Jarno Riistama, Fatima Sert Kuniyoshi, Theo Meyer, Robert Goldberg, David D McManus

**Affiliations:** ^1^ Philips Research Eindhoven Netherlands; ^2^ Department of Medicine University of Massachusetts Medical School Worcester, MA United States; ^3^ Department of Quantitative Health Sciences University of Massachusetts Medical School Worcester, MA United States; ^4^ Meyers Primary Care Institute University of Massachusetts Medical School Worcester, MA United States; ^5^ Department of Emergency Medicine University of Massachusetts Medical School Worcester, MA United States; ^6^ Philips Healthcare Murrysville, PA United States

**Keywords:** acute decompensated heart failure, rehospitalization, remote monitoring, transthoracic bioimpedance, wearable fluid accumulation vest

## Abstract

**Background:**

Recurrent hospital admissions are common among patients admitted for acute decompensated heart failure (ADHF), but identification of patients at risk for rehospitalization remains challenging. Contemporary heart failure (HF) management programs have shown modest ability to reduce readmissions, partly because they monitor signs or symptoms of HF worsening that appear late during decompensation. Detecting early stages of HF decompensation might allow for immediate application of effective HF therapies, thereby potentially reducing HF readmissions. One of the earliest indicators of HF decompensation is intrathoracic fluid accumulation, which can be assessed using transthoracic bioimpedance.

**Objective:**

The SENTINEL-HF study is a prospective observational study designed to test a novel, wearable HF monitoring system as a predictor of HF decompensation among patients discharged after hospitalization for ADHF.

**Methods:**

SENTINEL-HF tests the hypothesis that a decline in transthoracic bioimpedance, as assessed daily with the Philips fluid accumulation vest (FAV) and transmitted using a mobile phone, is associated with HF worsening and rehospitalization. According to pre-specified power calculations, 180 patients admitted with ADHF are enrolled. Participants transmit daily self-assessments using the FAV-mobile phone dyad for 45 days post-discharge. The primary predictor is the deviation of transthoracic bioimpedance for 3 consecutive days from a patient-specific normal variability range. The ADHF detection algorithm is evaluated in relation with a composite outcome of HF readmission, diuretic up-titration, and self-reported HF worsening (Kansas City Cardiomyopathy Questionnaire) during a 90-day follow-up period. Here, we provide the details and rationale of SENTINEL-HF.

**Results:**

Enrollment in the SENTINEL-HF study is complete and the 90-days follow-up is currently under way. Once data collection is complete, the study dataset will be used to evaluate our ADHF detection algorithm and the results submitted for publication.

**Conclusion:**

SENTINEL-HF emerged from our long-term vision that advanced home monitoring technology can improve the management of chronic HF by extending clinical care into patients’ homes. Monitoring transthoracic bioimpedance with the FAV may identify patients at risk of recurrent HF decompensation and enable timely preventive measures.

**Trial Registration:**

Clinicaltrials.gov NCT01877369: https://clinicaltrials.gov/ct2/show/NCT01877369 (Archived by WebCite at http://www.webcitation.org/6bDYl0dGy)

## Introduction

Hospitalizations related to acute decompensated heart failure (ADHF) have increased over recent decades [1], and ADHF is now the leading cause of hospital admissions in elderly patients [2]. In light of the high cost related to inpatient treatment of ADHF [3], there is increasing interest in deploying telehealth technologies to extend clinical care into the homes of patients with heart failure (HF), thereby allowing for more frequent assessment for and prevention of HF decompensations. However, the identification of HF patients in the early stages of decompensation remains a major clinical challenge. Significant efforts have focused on developing HF management programs to identify and intervene upon patients with or at-risk for ADHF in order to decrease HF-related hospitalizations [4-7]. Contemporary HF management programs rely on general, medication-related, and disease-specific patient education as well as active surveillance for signs or symptoms of acute HF decompensation. The impact of such programs has been limited, in part due to their cost, but also because HF surveillance tools, including measurements of heart rate, blood pressure, and body weight, show only modest abilities to identify individuals at risk for HF decompensation. Developing a home-based monitoring system using markers that can detect ADHF in its early stages is not only acceptable to elderly patients and facilitates communication between clinicians and patients, but has the potential to reduce rates of HF decompensation and hospitalization.

Novel measures of bioimpedance, or opposition to electric current through body tissues, can be used to identify fluid accumulation [8]. Prior studies have demonstrated that intrathoracic bioimpedance, measured in HF patients with implantable cardioverter-defibrillators, is a valid predictor of clinical events including ADHF and hospitalization [9]. Nevertheless, few patients with HF meet criteria for implantation of implantable cardioverter-defibrillators. More generalizable and less invasive methods to measure bioimpedance would capture a more representative population of patients with HF. Early data suggest that transthoracic bioimpedance measured using a wearable, investigational device may also detect intra-thoracic volume retention [10,11] and correlate with intrathoracic measures [12].

In order to explore the hypothesis that transthoracic bioimpedance assessed daily with a novel, non-invasive, wearable fluid accumulation vest (FAV) and transmitted using a mobile phone would identify patients at risk for HF related rehospitalization, we designed SENTINEL-HF, a prospective study of patients discharged after hospitalization for ADHF. Here, we describe the design and rationale of SENTINEL-HF.

## Methods

### Device Description

**Figure 1:**
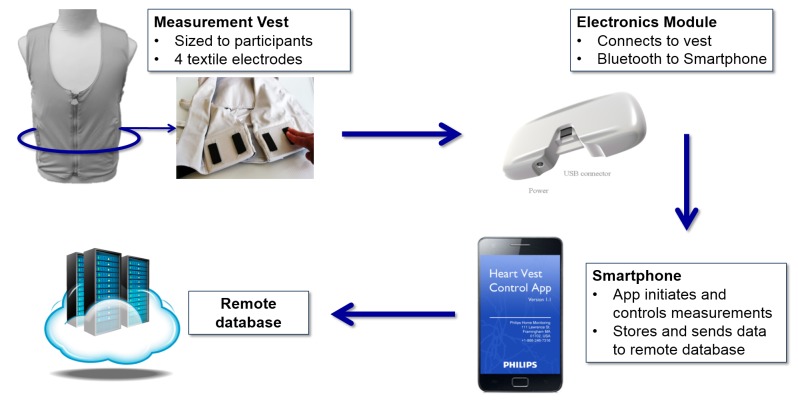
Components of the fluid accumulation vest (FAV) measurement system and the process for data acquisition, transfer, and storage.

The FAV is a non-invasive wearable monitor designed to spot-check transthoracic bioimpedance and send the measurement data to a remote telehealth center. The FAV system consists of a measurement vest, an electronics module, a mobile phone-based app, and a remote database (Figure 1). The measurement vest is a functional textile that is fitted snugly to the patient’s chest circumference using an adjustment strap and enables at-home self-measurements [13]. The inside of the vest has 4 textile electrodes arranged pair wise on 2 supporting pads located on either side of the rib cage, at the inferior part of the lungs. The electronics module connects to the back of the vest. The module determines transthoracic bioimpedance at multiple frequencies, which enables a model-based assessment of intra- and extra-cellular fluid status within the thorax [14] and the detection of respiration [15]. The module also records a 1-lead ECG as well as the patient’s motion and posture. The electronics module communicates wirelessly with a mobile phone-based app that guides the user through the measurement steps, controls the measurement parameters, and receives the measurement data. After each measurement, the app automatically transmits the data to a remote database hosted by the sponsor in a secure data center.

**Figure 2:**
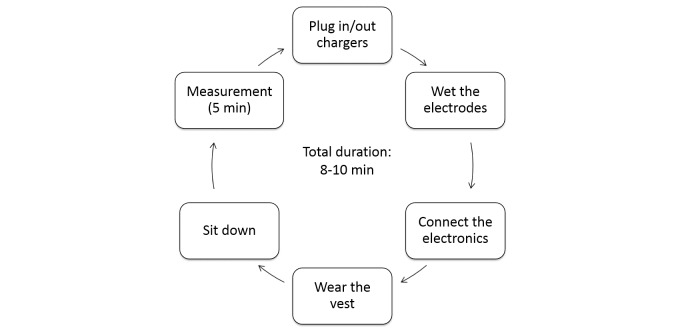
The daily measurement routine consists of wetting the electrodes (eg, using wetted fingertips), connecting the electronics module to the measurement vest, wearing the vest, sitting down, initiating a measurement with the mobile phone-based app, remaining seated for 5 minutes while the measurement is in progress, taking the vest off, and docking the devices for charging until the next day’s measurement.

Self-assessments with the FAV are performed by following a simple measurement routine that takes 8-10 minutes (Figure 2).

### Aim and Design

The aim of the SENTINEL-HF study is to evaluate whether transthoracic bioimpedance assessed daily using the FAV system following discharge from an HF hospitalization can predict recurrent HF decompensation events. The study also aims to assess patient adherence to the FAV system and delineate adherence profiles based on novel patient demographic and psychosocial characteristics.

SENTINEL-HF is a prospective, non-randomized, observational study. Patients are enrolled during a baseline hospitalization for HF and are monitored for 45-days post-discharge by means of daily self-assessments with the FAV. Patient-reported data related to healthcare utilization and quality of life are collected through interviews conducted during the baseline hospitalization and throughout the home monitoring period, at days 7, 14, and 45. Passive surveillance of participants for clinical events is performed through medical record review for 90 days following discharge. The extended passive surveillance period enables the examination of bioimpedance trends in relation with long-term outcomes.

Approximately 180 patients are enrolled at 2 teaching hospitals that comprise the University of Massachusetts Memorial Medical Center (UMMMC), a large academic medical center in Central Massachusetts. The UMMMC serves a heterogeneous patient population with a diverse ethnic/racial, socioeconomic, urban/rural, and other socio-demographic background. Medicare 30-days readmission and death rates for patients discharged from an HF-related hospitalization at UMMMC are similar to the US national rates (23% and 12%, respectively) [16].

### Study Population

The SENTINEL-HF study enrolls patients in New York Heart Association (NYHA) functional classes II-IV who are hospitalized with a primary diagnosis of HF or with a sign (ie, vascular congestion on chest radiograph, rales on lung exam, or peripheral edema) and a symptom (ie, dyspnea or orthopnea) consistent with ADHF. Adult patients (≥21 years) are eligible to participate if they are willing and able to comply with the clinical investigation plan, in particular, able to handle the technical devices, and are available for follow-up visits throughout a 45-days post-discharge period. Exclusion criteria include an N-terminal pro-brain natriuretic peptide (NT-proBNP) level ≤100 pg/dL, end-stage chronic kidney disease requiring hemodialysis, chronic obstructive pulmonary disease (COPD), a primary diagnosis of gastrointestinal bleed, asthma, cardiac arrest or acute coronary syndrome at baseline, primary pulmonary hypertension, psychiatric or neurological disorders of moderate to severe degree, non-English speakers, pregnant women, prisoners, patients planning to move from their residence within 2 months from baseline, a body habitus that prevents the patient from fitting into a measurement vest or does not allow for adequate electrode-skin contact while wearing the vest, non-intact skin at the location of the electrodes, or the presence of an implantable pacemaker/defibrillator.

### Endpoints

The ability of the FAV system to predict HF decompensation events is evaluated in relation to the composite endpoint of unplanned HF-related rehospitalization and HF worsening. A rehospitalization is defined as a presentation to the emergency department or any unplanned admission into a hospital environment with at least one overnight stay. HF worsening is defined as either diuretic up-titration or HF-related worsening quality of life, as assessed by the Kansas City Cardiomyopathy Questionnaire [17]. Secondary endpoints of the study include major adverse cardiac events, emergency department visits, all-cause rehospitalization, and death.

### Study Procedures

**Figure 3:**
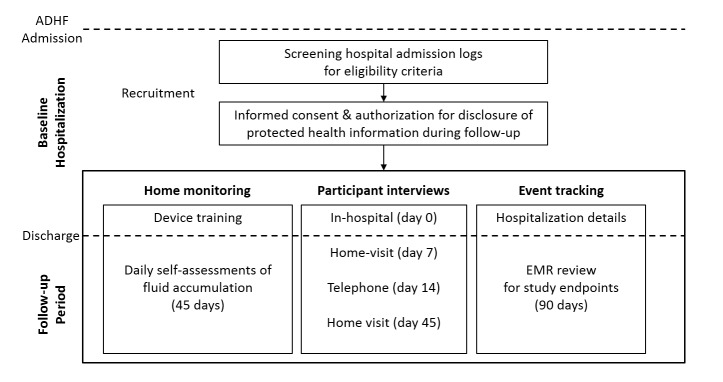
Diagram of the SENTINEL-HF study. This is a prospective, observational study in patients discharged after a baseline hospitalization for acute decompensated heart failure (ADHF). Participants will be recruited during the baseline hospitalization and follow-up activities will be initiated before discharge. Follow-up consists of 3 pillars: home monitoring which includes a period of self-assessments with the fluid accumulation vest (FAV), participant interviews, and event tracking based on participants’ electronic medical records (EMR).

SENTINEL-HF’s rich data collection is focused on 3 main activities (home monitoring, interviews, and event tracking), which are initiated during the baseline hospitalization and continued up to 90-days post-discharge. The study procedures are summarized in Figure 3 and are detailed in the following sections. Data collected during the study is outlined in Table 1.

**Table 1:** Data collected in SENTINEL-HF.

Domains and measures		Date of collection				
		Baseline	Day 7	Day 14	Day 45	Day 90
**Demographics**						
	Date of birth	X				
	Gender	X				
	Marital status	X				
	Race/ethnicity	X				
	Height	X				
	Weight	X				
	Thorax circumference	X				
**Baseline hospitalization details**						
	Chief complaint at admission	X				
	ICD-9 codes of hospitalization (first 3)	X				
	Medical histories and comorbidities	X				
	Type of cardiac dysfunction	X				
	Type of ventricular failure	X				
**Clinical events and outcomes**						
	Readmissions		X	X	X	X
	Emergency room visits		X	X	X	X
	Outpatient encounters		X	X	X	X
	Medication changes	X	X	X	X	X
**Patient reported outcomes: transition quality**						
	Care Transitions CTM-319		X	X	X	
**Patient reported outcomes: quality of life**						
	SF-12v2™ Health Survey [18]	X	X		X	
	Kansas City Cardiomyopathy Questionnaire (KCCQ) [17]	X	X	X	X	
	Self-Care of Heart Failure Index [19]	X	X	X	X	
**Psychosocial characteristics**						
	Capacity of informed consent [20]	X				
	Telephone interview for cognitive status (TICS) [21]	X	X	X	X	
	Patient Health Questionnaire (PHQ-9) [22]	X	X		X	

### Baseline Procedures

Patients with possible ADHF are identified using a computerized patient tracking system in the emergency department and by monitoring hospital admission logs on a daily basis. Screening is performed based on the diagnosis, chief complaint, and medical history. The eligibility of potential participants is confirmed based on their medical record information including laboratory records, ECG reports, and physical examination findings. Eligible patients are approached during hospitalization and provided with information about the study. Patients who agree to participate sign an informed consent and an authorization for disclosure of protected health information during the follow-up period (eg, details of subsequent readmissions). If study staff have concerns about the capacity of a potential participant to provide informed consent, an assessment [20] is conducted to determine the patient’s ability to express choice, to understand relevant information, and to appreciate the situation and its likely consequences. Patients who fail the assessment instrument are ineligible for the study.

Shortly before discharge, each enrolled participant is provided with a personal FAV monitoring kit consisting of a measurement vest of appropriate size, an electronics module, a study dedicated mobile phone, a manual of operation, and a short form instructions and troubleshooting guide. Staff adjust the measurement vest to the participant’s chest circumference ensuring that the electrodes have good contact with the participant’s skin, and train participants, and their caregivers when applicable, to use the study equipment.

### Home Monitoring

During a 45-day home monitoring period, participants are required to perform daily self-assessments with the FAV at a consistent time of day, preferably upon waking up in the morning. Study staff use scheduled follow-ups to ensure that the self-assessments are carried out correctly and, if necessary, to provide assistance in the form of additional instructions for using the FAV. On day 1, staff call each enrolled participant to check on their experience with the first FAV self-assessment. On day 7, study staff visit the participants at home providing a booster training for the use of the FAV system. On day 14, study staff call the participants encouraging them to continue with the daily self-assessments throughout the following month (Figure 3). Participants who require additional assistance at any time during the home monitoring period may call a study hotline maintained by the sponsor (Philips Healthcare).

Participants’ adherence to the daily self-assessment routine is monitored based on the data transmitted to the remote database. Study staff call participants with 2 consecutive missed or non-evaluable days to identify problems and to provide support in resuming the daily self-assessments. If 2 consecutive missed or non-evaluable days continue to occur after the call, study staff visit the struggling participants at home to troubleshoot technical issues and to walk the participants through the FAV self-assessment routine. Participants are removed from the study if 2 consecutive missed or non-evaluable days continue to occur after the home visit.

### Participant Interviews

In order to identify characteristics associated with the acceptance and the successful use of the FAV, participant interviews are conducted in-person or over the telephone at baseline and at day 7, 14, and 45 post-discharge (Figure 3). During these follow-up interactions, key patient reported outcomes and covariates, including severity of depressive symptoms and HF self-care are assessed. An overview of collected data and instruments used in the interviews is provided in Table 1.

At each follow-up interview, hospital-to-home transition quality is assessed using the 3-item Care Transition Measure (CTM3), an assessment designed to measure the quality of care transitions from the patient’s perspective [23]. The measure has good psychometric properties and has predictive validity for rehospitalizations. We also assess for HF self-management, patient response to changes in symptoms of HF, and confidence with self-management using the validated Self-Care for Heart Failure Index [19]. We measure both general and disease-specific quality of life at baseline and during each follow-up interview. General quality of life is measured by using the Short Form Health Survey SF-12, a validated 12-item version of the SF-36, a quality of life instrument often used for patients with heart disease [18]. HF-specific quality of life is assessed using the Kansas City Cardiomyopathy Questionnaire (KCCQ) [17].

Each participant’s psychosocial profile is characterized for a better understanding of factors associated with successful FAV use. Cognitive impairment and depressive symptoms are assessed at baseline and during each follow-up interview using the telephone interview for cognitive status (TICS) [21] and the 9-item Patient Health Questionnaire (PHQ-9) [22], respectively. We also gather information about clinical endpoints, including medication changes, doctor’s visits, emergency department visits, or readmissions. These reports are confirmed and extended by reviewing each participant’s medical record.

### Baseline Clinical Characteristics and Endpoint Review

Clinical information (medical history, physical exam data, chest x-ray findings, and serum laboratory values) and demographics (age, gender, marital status, race/ethnicity, height, weight, and blood pressure and heart rate) are abstracted at baseline from the participants’ electronic medical records. Surveillance of participants continues for 90 days post-discharge by reviewing all additional clinical data including hospitalizations or physician visits related to possible ADHF, cardiac studies, consultations, discharge summaries, emergency service records, lab reports, office and clinic notes, operative and procedure reports, problem lists, pulmonary studies, and radiology results from the electronic medical records. Because the majority (80-90%) of patients hospitalized for ADHF at UMMMC follow-up with a UMMMC cardiologist and are rehospitalized at UMMMC, the vast majority of participant data is available in one centralized database of electronic medical records. Information related to patient-reported clinical events occurring outside the UMMMC health system is obtained from the respective healthcare provider based on the participant’s consent for solicitation of outside records. Endpoints are adjudicated on a rolling basis by 3 expert physicians (authors TEM, DDM, CD) blinded to FAV measures and clinical outcomes.

### Data Analysis

The study database is used to evaluate the performance of a previously developed ADHF detection algorithm informed by bioimpedance measures.

#### Parameter Extraction

**Figure 4:**
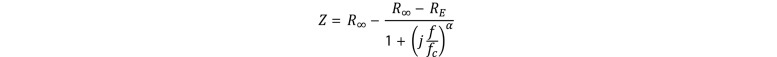
Cole-Cole model equation.

Relevant tissue characteristics are extracted from each FAV self-assessment by fitting the Cole-Cole model (Figure 4) to the multifrequency bioimpedance data collected by the device [14,24]. The model parameters reflect extracellular fluids (*R_E_*), intracellular fluids (*R*_*∞*_), tissue relaxation (*fc*), and tissue heterogeneity (*α*). Thoracic fluid accumulation due to worsening HF directly affects the value of *R_E_* which is expected to decrease [8,11]. Therefore, *R_E_* will be used as the primary bioimpedance index to predict HF decompensation.

#### Acute Decompensated Heart Failure (ADHF) Detection

**Figure 5:**
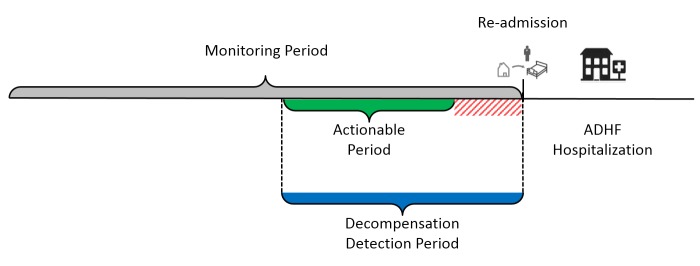
The ADHF detection strategy. Example of an ADHF hospitalization occurring during the FAV monitoring period. The ADHF detection algorithm is evaluated based on its ability to raise decompensation alerts within an actionable period which ends days prior to the event.

An ADHF detection algorithm was designed to monitor the daily evolution of a patient’s bioimpedance index. The algorithm determines if a new value is normal by comparing it to a patient-specific range of normal variability. The occurrence of abnormal values of the bioimpedance index causes the algorithm to raise decompensation alerts. In a real-life use case, decompensation alerts have the potential to prevent HF events, for example hospitalizations, only if they are raised a sufficient number of days prior to an acute decompensation allowing sufficient time for healthcare providers to intervene and for administered therapies to take effect. Because telehealth alerts are generally reviewed during business hours on weekdays, the reaction to an alert may be delayed by 2-3 days if an alert is raised at the beginning of a weekend. To accommodate for this in the evaluation of the ADHF detection algorithm we define the actionable period (AP) as the time window preceding an endpoint in which decompensation alerts are most likely to contribute to an improved patient outcome. The algorithm is evaluated based on its ability to raise alerts within the AP and any alert raised after the AP is excluded from the performance evaluation (Figure 5). The length and the end of the AP can be set by each healthcare provider individually. While healthcare providers generally agree that a realistic length for the AP is likely to be shorter than 30 days, setting the end of the AP remains a topic of debate. Therefore, we set the boundaries of the AP based on the performance achieved by current standard of care telehealth systems which include measurements of weight, heart rate, or blood pressure. These systems typically use weight as the primary predictor of decompensation and have the ability to raise decompensation alerts 2-3 days prior to an impending HF-related hospitalization [25]. In the analysis of the SENTINEL-HF study, we evaluate whether the FAV is able to extend this period by enabling earlier decompensation alerts. To this aim, we vary the end of AP down to 2 days prior to an endpoint event and the length of the AP between 10 and 30 days.

The predictive accuracy of the ADHF detection algorithm is assessed based on the number of true positive, true negative, false positive and false negative alerts preceding an endpoint. Prediction performance metrics are assessed in relation with the primary and the secondary endpoints, respectively.

### Sample Size Calculations

Our prospective sample size calculations were guided by the a priori assumption that the FAV would have a sensitivity of 0.75 to detect a primary endpoint of ADHF hospitalization. This assumption was based on a previous dataset collected for the development of our ADHF detection algorithm [12]. In SENTINEL-HF, we plan to have 107 patients complete the study period and adhere to the FAV daily over 45-days to exclude a real sensitivity lower than 0.60. To account for non-evaluable data and a 30% dropout rate, we require a minimum of 160 participants to provide a 0.1 95% confidence interval around an observed sensitivity of 0.75. We are employing an adaptive study design with interim sample size re-assessments based on the observed drop-out rate, non-evaluable data, and the number of ADHF events.

### Ethical Conduct and Study Management

The SENTINEL-HF study is conducted in compliance with Good Clinical Practice and the principles outlined by the Declaration of Helsinki. The study has been approved by the Committee for the Protection of Human Subjects at University of Massachusetts Medical School with Institutional Review Board number H00001760. All study participants provide written informed consent at enrollment. The study is sponsored by Philips Healthcare and is monitored by the sponsor in compliance with the Code of Federal Regulations. Unanticipated adverse device effects are reported to the sponsor and to the Massachusetts Medical School Institutional Review Board. The study trial registration number is NCT01877369.

## Results

SENTINEL-HF enrolled the first patient in June 2013 and the last patient in April 2015. A total of 180 patients are enrolled in the study. Once the 90-days follow-up of the last patient is complete, the SENTINEL-HF dataset will be used to evaluate our ADHF detection algorithm and first results will be submitted for publication.

## Discussion

The SENTINEL-HF study is a prospective, non-randomized, observational study evaluating the ability of a wearable HF monitor to anticipate ADHF events. Patients perform daily self-assessments of transthoracic bioimpedance for 45 days following discharge from a HF hospitalization. Patient-reported quality of life are assessed using validated questionnaires and clinical events are abstracted from the patients’ electronic medical records. Endpoints adjudicated by a team of physicians are used to evaluate the performance of an ADHF detection algorithm informed by serial bioimpedance measurements. SENTINEL-HF supports our long term vision that advanced home monitoring technology can aid the management of chronic HF by extending clinical care into patients’ homes, thereby preventing HF decompensations and reducing hospitalizations.

## Abbreviations

ADHF: acute decompensated heart failure
AP: actionable period
FAV: fluid accumulation vest
HF: heart failure
UMMMC: University of Massachusetts Memorial Medical Center

## References

[1] Fang J, Mensah GA, Croft JB, Keenan NL (2008). Heart failure-related hospitalization in the U.S., 1979 to 2004. J Am Coll Cardiol.

[2] Lloyd-Jones D, Adams RJ, Brown TM, Carnethon M, Dai S, De SG, Ferguson TB, Ford E, Furie K, Gillespie C, Go A, Greenlund K, Haase N, Hailpern S, Ho PM, Howard V, Kissela B, Kittner S, Lackland D, Lisabeth L, Marelli A, McDermott MM, Meigs J, Mozaffarian D, Mussolino M, Nichol G, Roger VL, Rosamond W, Sacco R, Sorlie P, Roger VL, Stafford R, Thom T, Wasserthiel-Smoller S, Wong ND, Wylie-Rosett J, American Heart Association Statistics CommitteeStroke Statistics Subcommittee (2010). Heart disease and stroke statistics--2010 update: a report from the American Heart Association. Circulation.

[3] Goldberg RJ, Ciampa J, Lessard D, Meyer TE, Spencer FA (2007). Long-term survival after heart failure: a contemporary population-based perspective. Arch Intern Med.

[4] de Lusignan S, Wells S, Johnson P, Meredith K, Leatham E (2001). Compliance and effectiveness of 1 year's home telemonitoring. The report of a pilot study of patients with chronic heart failure. European Journal of Heart Failure.

[5] Fonarow GC, Stevenson LW, Walden JA, Livingston NA, Steimle AE, Hamilton MA, Moriguchi J, Tillisch JH, Woo MA (1997). Impact of a comprehensive heart failure management program on hospital readmission and functional status of patients with advanced heart failure. Journal of the American College of Cardiology.

[6] Giordano A, Scalvini S, Zanelli E, Corrà U, Longobardi GL, Ricci VA, Baiardi P, Glisenti F (2009). Multicenter randomised trial on home-based telemanagement to prevent hospital readmission of patients with chronic heart failure. Int J Cardiol.

[7] Hall P, Morris M (2010). Improving heart failure in home care with chronic disease management and telemonitoring. Home Healthc Nurse.

[8] Weyer S, Zink MD, Wartzek T, Leicht L, Mischke K, Vollmer T, Leonhardt S (2014). Bioelectrical impedance spectroscopy as a fluid management system in heart failure. Physiol Meas.

[9] Ypenburg C, Bax JJ, van der Wall EE, Schalij MJ, van Erven L (2007). Intrathoracic impedance monitoring to predict decompensated heart failure. Am J Cardiol.

[10] Cuba-Gyllensten I, Gastelurrutia P, Riistama J, Aarts R, Nuñez J, Lupon J, Bayes-Genis A (2014). A novel wearable vest for tracking pulmonary congestion in acutely decompensated heart failure. Int J Cardiol.

[11] Dovancescu S, Torabi A, Mabote T, Caffarel J, Kelkboom E, Aarts R, Korsten E, Cleland J (2013). Sensitivity of a wearable bioimpedance monitor to changes in the thoracic fluid content of heart failure patients. http://ieeexplore.ieee.org/xpl/articleDetails.jsp?&arnumber=6713530.

[12] Harris M, Habetha J (2007). The MyHeart project: a framework for personal health care applications. http://www.cinc.org/archives/2007/pdf/0137.pdf.

[13] Reiter H, Muehlsteff J, Sipilä A (2011). Medical application and clinical validation for reliable and trustworthy physiological monitoring using functional textiles: Experience from the HeartCycle and MyHeart project.

[14] Buendia R, Gil-Pita R, Seoane F (2011). Cole parameter estimation from the modulus of the Electrical Bioimpeadance for Assessment of Body Composition. A full spectroscopy approach. J Electr Bioimp.

[15] Dovancescu S, Para A, Riistama J (2014). Detection of electrocardiographic and respiratory signals from transthoracic bioimpedance spectroscopy measurements with a wearable monitor for improved home-based disease management in congestive heart failure. http://ieeexplore.ieee.org/xpl/articleDetails.jsp?&arnumber=7043210.

[16] Medicare Hospital Compare.

[17] Green CP, Porter CB, Bresnahan DR, Spertus JA (2000). Development and evaluation of the Kansas City Cardiomyopathy Questionnaire: a new health status measure for heart failure. J Am Coll Cardiol.

[18] Ware J, Kosinski M, Keller SD (1996). A 12-item short-form health survey: construction of scales and preliminary tests of reliability and validity. Med Care.

[19] Riegel B, Carlson B, Moser DK, Sebern M, Hicks FD, Roland V (2004). Psychometric testing of the self-care of heart failure index. J Card Fail.

[20] Schmitt EM, Marcantonio ER, Alsop DC, Jones RN, Rogers SO, Fong TG, Metzger E, Inouye SK (2012). Novel risk markers and long-term outcomes of delirium: the successful aging after elective surgery (SAGES) study design and methods. J Am Med Dir Assoc.

[21] Brandt J, Specter M, Folstein M (1988). The telephone interview for cognitive status. Neuropsychiatry Neuropsychol Behav. Neurol.

[22] Kroenke K, Spitzer R (2002). The PHQ-9: a new depression diagnostic and severity measure. Psychiatr. Ann.

[23] Parry C, Mahoney E, Chalmers SA, Coleman EA (2008). Assessing the quality of transitional care: further applications of the care transitions measure. Med Care.

[24] Cole KS, Cole RH (1941). Dispersion absorption in dielectrics I. Alternating current characteristics. J. Chem. Phys.

[25] Zhang J, Goode KM, Cuddihy PE, Cleland JG, Ten-HMS Investigators (2009). Predicting hospitalization due to worsening heart failure using daily weight measurement: analysis of the Trans-European Network-Home-Care Management System (TEN-HMS) study. Eur J Heart Fail.

